# Use of Integrative Interactomics for Improvement of Farm Animal Health and Welfare: An Example with Fescue Toxicosis

**DOI:** 10.3390/toxins12100633

**Published:** 2020-10-01

**Authors:** Ryan S. Mote, Nikolay M. Filipov

**Affiliations:** Interdisciplinary Toxicology Program, Department of Physiology and Pharmacology, University of Georgia, Athens, GA 30602, USA; ryan.mote25@uga.edu

**Keywords:** integrative interactomics, integrome, metabolomics, microbiome, tall fescue, *Epichloë coenophiala*, fescue toxicosis

## Abstract

Rapid scientific advances are increasing our understanding of the way complex biological interactions integrate to maintain homeostatic balance and how seemingly small, localized perturbations can lead to systemic effects. The ‘omics movement, alongside increased throughput resulting from statistical and computational advances, has transformed our understanding of disease mechanisms and the multi-dimensional interaction between environmental stressors and host physiology through data integration into multi-dimensional analyses, i.e., integrative interactomics. This review focuses on the use of high-throughput technologies in farm animal research, including health- and toxicology-related papers. Although limited, we highlight recent animal agriculture-centered reports from the integrative multi-‘omics movement. We provide an example with fescue toxicosis, an economically costly disease affecting grazing livestock, and describe how integrative interactomics can be applied to a disease with a complex pathophysiology in the pursuit of novel treatment and management approaches. We outline how ‘omics techniques have been used thus far to understand fescue toxicosis pathophysiology, lay out a framework for the fescue toxicosis integrome, identify some challenges we foresee, and offer possible means for addressing these challenges. Finally, we briefly discuss how the example with fescue toxicosis could be used for other agriculturally important animal health and welfare problems.

## 1. Introduction

As world population continues to climb [1], improving production efficiency without sacrificing rigorous quality or safety standards is crucial for sustaining the agricultural supply chain [2]. Significant geopolitical and extreme weather events pose additional negative pressures, while consistent economic growth drives agricultural demand, leading to new challenges for producers and scientists alike [2]. Other extenuating circumstances, like increased risk of a global pandemic in our highly connected world, i.e., the Coronavirus disease 2019 (COVID-19) pandemic the world is currently faced with [3], also pose a threat to the global food supply. In the face of these challenges, maintenance of a sustainable, efficient, and adaptable agricultural supply chain is essential. Scientific advances have begun to increase the efficiency of outlining pathophysiological responses by generating high-volume, high-quality datasets quickly, providing novel biological targets for historical and future maladies that may have been missed when using more classical, low-throughput techniques [4]. Thus, recent scientific and technological advances, in conjunction with classical techniques, will continue to improve the ability of the agricultural sector to adapt to both predictable and unforeseen future challenges.

High-throughput technologies are changing the way we investigate complex biological systems by generating large data sets coupled with advanced computational and bioinformatics tools. This includes recent efforts towards combining multi-level data matrices (e.g., genomics, proteomics, metabolomics, etc.). The main focus of the current review is on the application of these technologies to agriculturally important, mycotoxin-induced diseases. 

Next-generation sequencing (NGS) can generate high-quality sequencing data regardless of sample origin, increasing our understanding of how genotype influences the ability of organisms to adapt to internal and external stressors. For example, NGS can screen plants for genes associated with agronomic benefits [5]. From a farm animal perspective, NGS has outlined the relationship between ruminant microbes and animal productivity [6,7], allowing investigation of complex ecosystems derived from NGS’ culture-independency. Improvements in sequencing technologies have also provided new insights into ecological shifts that occur in the host microbiota after different dietary/treatment regiments [8,9]. Culturomics, a new field centered on cultivating ruminant microorganisms, utilizes NGS and is driven by understanding the biochemical potential of individual microbes [10]. 

High-resolution mass spectrometry propelled the investigation of the proteome and metabolome [11]. Metabolomics, the study of all low molecular weight compounds within a biological matrix [12], can be used to examine biochemical interactions [13] or to further our understanding of food quality, processing and safety [14]. Much like NGS, advances in the metabolomics computational framework have increased the utility and capacity of metabolomics by improving detection and quantification of both prominent and low abundance metabolites without sacrificing quality [15,16,17,18]. Notably, metabolomics scalability has many potential uses; one of the most promising is for biomonitoring of environmental exposures, as suggested for deployed troops [19]. Although computational metabolomics has been proposed for evaluation of the human exposome for a detailed review on the exposome; see: [20], these advances are translatable for improving food safety/quality and, as detailed here, agricultural animal health/wellness.

**Figure 1:**
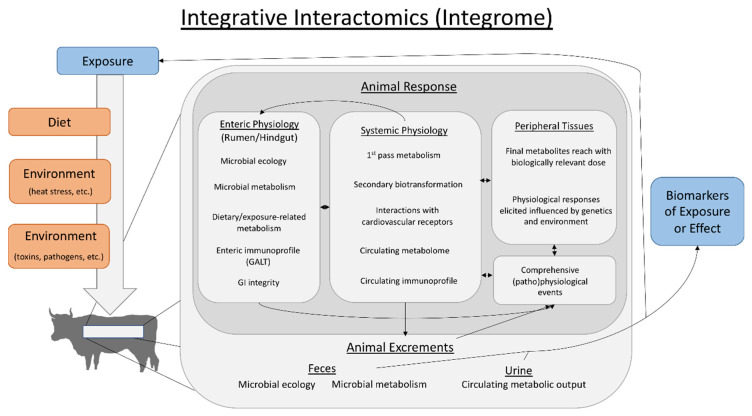
A general, adaptable schematic of the integrome as evaluated through integrative interactomics based on dietary/toxic exposures from an animal agricultural perspective. Animals are exposed to combinations of dietary components, ambient environmental conditions, and external toxicants or pathogens from the environment. Multi-‘omics data are collected and integrated within multi-compartment animal responses, which consists of evaluation of enteric (rumen and hindgut herein) and systemic physiological changes, understanding effects on peripheral tissues, and how this results in (patho)physiological effects of interest. Further, animal excrements are an ideal, readily accessible biological matrix that can be utilized to identify biomarker(s) of exposure and/or effect, including those associated with pathophysiological changes. Finally, evaluating how components of the excrements feed back into what animals are exposed to through their diet is also important to the integrome. Overall, this model can help outline global changes that occur in any situation through systematic evaluation of complex biological processes and interactions.

One important multi-‘omics development is computational tools that integrate different ‘omics data types (e.g., genome, proteome, metabolome, etc.) into single, interpretable outputs, allowing an evaluation of the integrome through integrative interactomics [21]. Traditionally, interactomics is a broad, yet well-established field focused on mRNA-protein [22] and/or protein-protein [23] interactions within a large spectrum of biological systems and disorders [24,25,26]. To contrast this view of the interactome, herein we have defined the integrome as the integration of multi-‘omics data sets through integrative interactomics, allowing for the description of systemic biological interactions and multi-organ-integrative responses to internal or external variables. The proposed generic framework for the integrome is presented in Figure 1, where grazing animals are used as the example. This initial iteration of the integrome has multiple components. First, molecular characteristics of dietary components are influenced by environmental factors, and a composition of dietary, environmental, and toxin-related exposures determine what animals are exposed to on a daily basis (Figure 1). In response to oral exposures, the animal’s enteric physiology shifts to adapt to dietary, environmental and toxicological pressures by altering the microbiome, microbial metabolites, and diet-derived metabolites. The enteric immunoprofile, metabolite/microbe interactions with gut associated lymphoid tissues (GALT), and potential effects on gut wall integrity further effect downstream (patho)physiology. Overall, these enteric physiological shifts dictate what metabolites are present within the gastrointestinal (GI) tract and are absorbed into systemic circulation. Further, the enteric immune and enteroendocrine systems signal and influence downstream physiological processes in peripheral tissues (Figure 1). Evaluating metabolites that enter circulation and their fate (e.g., metabolism, receptor interactions, etc.) in the larger biological system is a reflection on how diet and environmental exposures influence downstream host physiology (Figure 1). Finally, microbes and metabolites excreted from the animal, along with grazing and other environmental stresses, may influence, for example in pasture production settings, plant physiology, creating a feedback loop between the plant, dietary/environmental exposures and (patho)physiological changes within the animal (Figure 1). Notably, the excrements (fecal matter, urine) are easily accessible tools and can be used for investigating novel biomarkers of exposure or effect. This framework evaluates systemic, multi-organ responses to dietary and environmental stressors, while considering effects on the entirety of the diet-environment-animal interactions. Overall, technological and computational advances have opened new opportunities to evaluate global, systemic biological interactions. For a detailed review of the computational methods associated with multi-‘omics data integration, the mathematical concepts behind these methods, and their application towards toxicological risk assessment, which is outside the scope of the current paper, the readers are directed to the following reviews [27,28,29,30].

## 2. Technological Advances Increase Throughput for Toxicology Screening

The field of toxicology stands to benefit from integrative interactomics [30]. For example, the Toxicology in the 21st century (Tox21) joint venture in the United States is focused on increasing throughput for toxicity screenings (https://tox21.gov/). Drawing conclusions about how individual compounds or mixtures interact within a biological system (e.g., cell, organism) is difficult, but using top-down strategies (i.e., using high-volume global analyses to understand molecular signatures, such as ‘omics-based analyses) can provide valuable biological insights missed by bottom-up strategies. Moreover, top-down strategies can evaluate adaptive and pathophysiological responses in plants [31], humans [32], and model organisms [33], which is an important step in understanding the toxicity mechanisms and consequences of environmental exposures. Examples in this regard are low-dose chronic exposures to heavy metal and pesticide mixtures [34,35]. Although these approaches have been previously applied in different contexts, they have specific utility for livestock and large animal physiology/toxicology. Recent work highlighted the bovine genome-microbiome relationship [36], bovine microbiota response to dietary additives [37], and the use of host-microbiota relationship to establish more efficient production systems [38]. 

Mycotoxin exposures are a major livestock concern. Aflatoxins perturb the immune system [39] and rumen motility [40], while *Fusarium* mycotoxins (e.g., T-2 toxin) adversely impact livestock reproduction [41]. Reproductive and growth impairments, discussed in detail later, are also associated with *Epichloë* mycotoxins [42]. Integrative interactomics provides the opportunity to identify novel, mutli-level evidence of direct/indirect mycotoxin effects that, when coupled with traditional methods, can be exploited therapeutically.

## 3. Maintaining Animal Productivity and Wellness in Adverse Environments, Toxicity Included

Improving and maintaining farm animal productivity and wellness is critical. NGS has provided insights into the importance of host genetics on the bovine microbiota [38,43], which seems to be stable after rumen transplant in dairy cattle [44], and the association between microbial genes in the rumen and feed efficiency [45,46,47]. Further, how the microbiota is influenced by diets/dietary additives has been evaluated [48,49]. As shown recently, the ruminant microbiota is influenced mostly by diet, only partially by the host, and a core microbiota exists across geographies [50,51]. Moreover, rumen microbial communities are influenced by age and environment [52], indicating the developing microbiota could be important for diseases that are either heritable and/or have pre- or postnatal contributing factors. Overall, evaluating the enteric microbiota after exposure to dietary additives or environmental contaminants, as well as after disease onset, is important, since understanding the adaptive microbiota response to these adverse events can help provide a complete picture of the microbiota’s role in health and disease.

Metabolomics can also provide important insights into livestock productivity. For example, metabolomics-based methods were used to associate rumen fluid metabolites with feed efficiency [53], assess biochemical variability in bovine milk [54], and identify differences in the bovine milk metabolome during early and late gestation [55]. Metabolomics is used widely to search for biomarker signatures of feed efficiency and disease states in cattle. Livestock metabolomics is not major focus of this review; for an in-depth review, refer to Goldansaz, et al. [56].

How fungal mycotoxins/metabolites influence rumen microbial ecology in animals consuming contaminated feedstuffs is of major interest [57]. While ruminants are considered less susceptible to mycotoxins than monogastrics, ruminant mycotoxicoses do occur and are problematic for animal health and welfare, for a detailed review on ruminant mycotoxicosis, see [58]. Aflatoxin B1 (AB1) is a well-studied mycotoxin; its effects and/or potential biomarkers for exposure in dairy cattle plasma [59,60], as well as rumen fluid and milk [61], have been investigated with metabolomics-based methods. The non-ruminant (porcine) colonic microbiota, namely its functional biodiversity, is impacted by *Fusarium* mycotoxins [62]; however, despite this, and the utility of NGS and metabolomics in evaluating animal productivity, integrating these separate data sets towards understanding systemic biological responses and the livestock integrome is yet-to-be widely done. 

As a whole, animal agriculture would benefit greatly from the development, characterization, and utilization of integrative interactomics-based approaches, and this has been proposed for human health as well [63]. Although not in livestock, using an integrative metagenomics and metabolomics approach, Lu, et al. [64] reported that arsenic exposure in drinking water significantly perturbs the host (mouse) microbiota, metabolome, and multiple microbiota-related metabolites. A similar approach was also able to identify a novel relationship between the microbiota, certain metabolites and colorectal cancer, indicating a potential utility of microbial metabolites as diagnostic or therapeutic targets [65]. Finally, Tang, et al. [66] were able to identify microbiota and metabolites that were significantly influenced by diet, indicating that dietary constituents not only influence the gut microbiota, but have systemic effects as well. Similar investigations integrating microbiota and metabolomics data from multiple biological compartments in livestock could provide significant insights into the mechanisms by which dietary, environmental, and mycotoxin exposures influence animal performance. 

New data from our lab [67,68,69], among other recent reports [38,70,71], indicate that developing integrative interactomics-based approaches to complement other, more traditional methods for evaluating the livestock integrome could increase our understanding about the development and adaptive versus pathophysiological responses to one of the costliest diseases to the United States beef industry, fescue toxicosis. The rest of this review will focus on introducing what we currently know about fescue toxicosis development and pathophysiology and how newly generated ‘omics data could be integrated to help fill crucial knowledge gaps related to this disease. 

## 4. Fescue Toxicosis

### 4.1. Tall Fescue and Epichloë Coenophiala

Tall fescue, *Lolium arundinaceum*, is a cool season perennial grass that is well adapted to the Southeastern United States, covering approximately 14 million hectares of land. Tall fescue has many compelling agronomic attributes, such as persistence under drought and grazing stresses, resistance to herbivores, nematodes, and insects, and greater potential for nutrient uptake [72]. Ultimately, these attributes give it a competitive advantage over other cultivars and stem from an infection of the plant with the endophytic fungus *Epichloë coenophiala* [72].

*E. coenophiala* grows intercellularly and systemically above ground [73] and is vertically transmitted through tall fescue seed heads [74]. Interestingly, the agronomic benefits of *E. coenophiala* infection appear to be heritable, with both the plant and endophyte genotype influencing offspring persistence [75]. Endophyte-derived bioactive secondary metabolites are considered responsible for the increased persistence of *E. coenophiala*-infected tall fescue. While some metabolites are beneficial, one group, the ergot alkaloids, have been shown to be detrimental to grazing livestock, inducing the development of fescue toxicosis [76]. Ergot alkaloids are the most abundant class of bioactive secondary metabolites produced by *E. coenophiala* and are the ones of greatest economic concern in the US [42,77] and worldwide [78,79].

### 4.2. Ergot Alkaloids: Presence, Biosynthesis, and Monoaminergic Activities

The ergot alkaloid profile of tall fescue is variable and consists of lysergic acid derivatives and ergopeptine alkaloids. Both share the same pharmacophore (D-lysergic acid), but classification is derived from functional groups bonded to the primary 8th carbon [80]. Lysergic acid derivatives have a carboxamide functional group, with varying chemical entities bound here used for classification. The ergopeptines have a tripeptide moiety bound to carbon 8 and this is used for ergopeptine nomenclature. For example, the major ergopeptine alkaloid derived from *E. coenophiala*, ergovaline, has L-alanine, L-proline, and L-valine as constituents, whereas ergotamine, a *Claviceps purpurea* ergopeptine, has L-alanine, L-proline, and L-phenylalanine as constituents [77].

Studying the activities of ergot alkaloids in biological systems is difficult because the pharmacophore (i.e., the D-lysergic acid backbone) bears structural similarities with dopamine (DA), norepinephrine (NE), and serotonin [5HT; 80]. This allows ergot alkaloids to have systemic interactions, eliciting serotonergic, dopaminergic, and adrenergic activities that can influence numerous physiological functions [80]. 

### 4.3. Ergot Alkaloid Metabolism in Ruminant Animals

The pharmacokinetics of bromocryptine, a synthetic ergot alkaloid, have been extensively studied, but the toxicokinetics of the natural alkaloids of endophyte-infected tall fescue are not well characterized. That said, ergot alkaloids and their metabolites (i.e., lysergic acid) have been detected in the serum after direct injection [81], urine [82,83], bile [83], fluids of the rumen and abomasum [84], subcutaneous fat [85], and liver and kidney tissues [86]; outside the urine, in many instances, the levels are minute. The amount of ergot alkaloids detected decrease through sequential sampling along the GI tract, with one study finding 50–60% recovered from abomasal contents and 5% in the feces [87]. Further, as much as 93–96% of ergot alkaloids are excreted in the urine, although the main metabolite detected in the urine is lysergic acid, suggesting extensive metabolism [83]; lysergic acid is also the main metabolite that crosses gastric tissues [84]. Parent ergopeptine alkaloids are not transported across gastric tissues in vivo and are likely metabolized in the rumen, indicating that downstream processes may contribute to fescue toxicosis development [88]. Overall, these data indicate that ergot alkaloids undergo complex pre-systemic metabolism in the rumen. That said, the effects of ergot alkaloids and their metabolites on microbial populations and microbial metabolism, the potential downstream perturbations caused by this (e.g., microbial-mediated global metabolic effects, immune system effects, etc.), and when the animals transition from an adaptive response to a pathophysiological state need to be investigated; top-down, integrative interactomics-based approaches can help begin this journey.

### 4.4. Biomarkers of Ergot Alkaloid Exposure and Effect

Biomarkers are reproducible, quantifiable biological constructs that result from biological activities and can be used to identify different medical or disease states [89]. Biomarkers need to have high reproducibility across studies or trials while being able to encapsulate the entirety of what it is they are being used to identify. Generally speaking, there are two types of common biomarkers: (1) biomarkers of exposure and (2) biomarkers of effect [90]. Biomarkers of exposure are biological indicators that exposure to a particular substance or compound of interest has occurred, whereas biomarkers of effect indicate that, generally, a pathological effect or disease state is present within a given system. While many biomarkers for fescue toxicosis have been proposed, evidence suggests most of the proposed biomarkers are biomarkers of exposure. For example, it has previously been shown that if ergot alkaloids cross gastric tissues into systemic circulation, they can interact with dopamine receptor subtype 2 (DRD2) receptors on anterior pituitary lactotrophs to decrease serum prolactin [91]. Decreases in serum prolactin have been proposed as a biomarker for ergot alkaloid exposure, but prolactin levels are known to be influenced by photoperiod [92], acute ambient temperature changes [93], and different forms of stress [94,95], which could all lead to reproducibility problems. Detection of total ergot alkaloids in the urine is an accurate, less variable method to assess ergot alkaloid exposure when compared to previous methods, such as decreased serum prolactin [82,83]. This is because ninety-four percent of ergot alkaloid excretion occurs in the urine, appearing as early as 12 h post-exposure, and concentrations are exposure level- and duration-dependent [82]. While urinary alkaloids are of great utility as a sensitive and reproducible biomarker of ergot alkaloid exposure and correlate with reduction in average daily weight gains [ADG; 82], this approach does not speciate individual ergot alkaloids or their metabolites. Speciating ergot alkaloids and metabolites, in plasma, urine, or rumen fluid, might help identify how ruminants metabolize ergot alkaloids and other metabolic processes that may be affected [82,84], which is where integrating multi-‘omics data sets will be beneficial. 

### 4.5. Adverse Effects of Toxic Tall Fescue Grazing and Ergot Alkaloids on Livestock

Despite notable stand persistence, which sparked initial interest in tall fescue as a pasture cultivar, reports regarding the detrimental impact of tall fescue grazing in livestock began as early as the 1940’s [96,97,98]. While cattle grazing tall fescue exhibit numerous signs, such as fescue foot, thermoregulatory impairment, and decreased feed intake, the most economically costly are lowered weight gains and reproductive insufficiencies [99]. 

Decreased muscle accretion and weight gains are common findings in steers grazing toxic tall fescue. This is a major concern in the beef industry, as these effects can go unnoticed despite their financial impacts [99]. Weight gains are decreased, in part, by lower feed intake and shortened grazing periods; these signs are exacerbated under hot and humid environmental conditions [i.e., heat stress; 99]. The molecular mechanism(s) that lead to decreased food intake are unknown, but current investigations are attempting to elucidate these mechanisms. Considering the dopaminergic and serotonergic activities of ergot alkaloids, it is plausible that one mechanism could be through alterations of gut motility. Strickland, et al. [100] were some of the first to suggest that interaction between ergopeptine alkaloids and enteric receptors [101] could potentially influence gut motility and feed intake. Though the authors noted the interactions between ergot alkaloids and enteric DRD2 receptors, we now know enteric serotonergic activities of ergot alkaloids are another plausible source for altered gut motility induced by toxic tall fescue exposure [101,102]. In support, increases in the baseline tonus of the rumen/reticulum and increased amplitude of reticular contractions follow intravenous infusion of ergovaline, the major ergopeptine in tall fescue [103]. Of note, increased rumen fill that could not be explained by increased dry matter intake, indicate ruminal passage rates may be decreased [104,105,106,107] in the absence of changes in energy balance (e.g., energy intake, O_2_ consumption, CO_2_ production, heat production, etc.) or feed digestibility [105]. Moreover, voluntary dry matter intake can be inhibited by increases in rumen fill and restricted flow of digesta to the lower gastrointestinal tract [108]. This indicates that, although digestibility and basal metabolic rates may not be affected, changes in gastrointestinal physiology post ergot alkaloid exposure, among other specific metabolic changes (i.e., certain metabolic pathways), could contribute to decreased muscle accretion and weight gains associated with fescue toxicosis through molecular changes. Although these data refer to direct actions of ergot alkaloids, most ergot alkaloids are degraded in the rumen [88]. Therefore, studies investigating the mechanisms behind the decreased weight gains and altered feeding behaviors need to consider: (1) ergot alkaloid metabolites that could directly influence host physiology and (2) indirect effects of ergot alkaloids and their metabolites.

## 5. The Case for Integrative Interactomics in Fescue Toxicosis Studies

Much is known about specific effects of toxic tall fescue grazing and ergot alkaloid exposure in the bovine, but there are still gaps of knowledge about fescue toxicosis pathophysiology. While there is a need for studying specific effects and/or responses, there is also an urgent need to examine the entire system as one integrated set of responses that contribute to decreased animal performance. First, from a plant perspective, evaluating global effects of *E. coenophiala* infection on important plant physiological characteristics (e.g., rhizosphere, phyllosphere, and endosphere microbiota, the plant metabolome, root exudates, etc.) will provide deeper insights into endophyte-derived molecular changes in the plant that animals are exposed to. Further, top-down strategic approaches may help identify global plant responses, outside of fungal ergot alkaloids, that could contribute to fescue toxicosis pathophysiology. If we assume ergot alkaloids, or the ergot alkaloid pharmacophore, enter the bloodstream and reach target receptors, their receptor promiscuity would result in complex physiological changes. The complexity of studying fescue toxicosis is derived from having to understand the plant-endophyte relationship growing in different geographical regions alongside direct and indirect effects of the entire tall fescue plant, endophyte, and endophyte-derived compounds on the grazing animal. However, an integrative interactomics-based approach would provide multi-level global analysis of different compartments between the plant and animal, providing a breadth of data that begins to address the true complexity of the disease by providing unique integrated information about multi-levels effects of *E. coenophiala.* When these are coupled with specific analyses of production parameters (i.e., weight gain) and/or physiological measurements (e.g., respiration rates), integrative interactomics will help with the identification and prioritization of therapeutic and management strategies of the disease. The complex relationship between the soil, plant, endophyte, and animal is the ideal case for studying the integrome through such systems biology-based approaches. The rest of this review will focus on how systems biology-based approaches have been used previously and demonstrate why integrative interactomics should be used to evaluate the fescue toxicosis integrome and use it to improve disease management alongside current methodologies. 

### 5.1. Effects of Epichloë Coenophiala Infection on the Plant

Felitti, et al. [109] performed transcriptome analyses on *Neotyphodium* (*Epichloë*) *coenophiala*, *Neotyphodium lolii*, and *Epichloë festucae*, and were able to compare functional changes in expressed sequence tag (EST), a subsequence of cDNA, resulting from growing these fungi on different media, indicating endophyte selection may result in functional in planta changes. Another study found that the endophytes that infect tall fescue plants play a significant role in modulating the rhizosphere and root exudate and how those secretions are modified by soil microbes, indicating a potential utility for endophyte selection to manipulate soil qualities [110]. In support, Rojas, et al. [111] noted that *E. coenophiala* infection significantly impacts the soil and rhizosphere fungal community population, with similar effects being seen for all endophytes tested. From another perspective, Rasmussen, et al. [112] have published a review summarizing their work, where they’ve assessed the effects of *Neotyphodium lolii* infection on the *Lolium perenne* plant metabolome. One difficulty of this approach is assessing what changes in the plant metabolome may be associated with biotransformation of endophytic metabolites, but Rasmussen and colleagues outlined a potential network of metabolites that are highly integrated between the endophyte and the plant, indicating complex plant-endophyte cross talk [112]. In support, a meta-analysis indicated that *Epichloë* endophytes promote resistance to insects through certain alkaloids and jasmonic acid pathway metabolites Bastias, et al. [113]. Altogether, these data indicate that endophyte infection significantly alters the rhizosphere and plant microbiota and metabolome, via an incredibly complex plant-endophyte relationship. However, no study has combined plant data in the same study that assesses animal responses to toxic tall fescue grazing. 

### 5.2. Effects of E. coenophiala-Infected Tall Fescue on the Animal

#### 5.2.1. Toxic Tall Fescue Effects along the Bovine Alimentary Tract 

The fate of ergovaline along the enteric tract was previously investigated [88]; it was undetectable in rumen fluid or urine in both in vitro and in vivo experiments. Only lysergic acid was able to cross rumen and omasal tissues, indicating that parent ergopeptine alkaloids likely do not make it into circulation at significant quantities through enteral absorption without undergoing metabolism/biotransformation. That said, one possible route that has yet to be thoroughly explored is possible ergot alkaloid absorption through the lymphatic system. The initial parts of the lymphatic system rely on peristalsis as one mechanism to fill and empty [114]. Considering ergot alkaloids can influence peristalsis through interactions with receptors in the enteric smooth muscle [100,101,102,103], ergot alkaloids could be absorbed through the lymphatic networks of the intestinal smooth muscle [115] while also influencing the ability of the initial lymphatic system to fill and empty. However, this possibility needs to be explored further. Overall, these data, combined with Section 4.3., point towards indirect mechanisms through which ergot alkaloids induce fescue toxicosis. Thus, understanding direct, indirect, and systemic effects of toxic tall fescue, ergot alkaloids and their metabolites (e.g., lysergic acid) is crucial to deciphering fescue toxicosis pathogenesis and progression.

To date, limited studies exist examining the effects of *E. coenophiala* on the bovine microbiota. The fungus *Aspergillus terreus* was consistently found along the enteric tract in steers that exhibited signs of fescue toxicosis (e.g., fescue foot), indicating enteric fungi may play a role in fescue toxicosis etiology or be useful as a biomarker [116]. Clavine alkaloids, which are ergot alkaloid precursors, appear to have antibiotic-like properties [117,118]. Previous work has also shown that ewes inoculated with an antibiotic resistant *Escherichia coli O157:H7* and fed a high endophyte-infected, versus low endophyte-infected seed shed a greater number of antibiotic resistance genes [119]. Recently, Harlow, et al. [120] investigated rumen bacteria that may degrade ergopeptine alkaloids by evaluating hyper-ammonia producing bacteria (HAB) and tryptophan-utilizing bacteria in vitro; they found that all tested HAB (e.g., *Clostridium* spp.) degraded ergovaline, with varying degrees of effect. Another recent study [71] assessed the effects of tall fescue seed and red clover isoflavones on global rumen microbial populations in vitro and found limited effects of tall fescue seed on volatile fatty acids. Interestingly, significant effects on bacteria aligned within the *Ruminococcaceae*, *Coriobacteriaceae*, and *Erysipelotrichaceae* families, which is similar to results from our lab where we saw E+ exposure altered the abundances of operational taxonomic units (OTUs) aligned to these families in vivo [67]. Most importantly, we found that toxic tall fescue grazing steers had a unique fecal microbiota structure that could provide insights into easily accessible biomarkers of exposure or effect. In another study, we found similar effects on the fecal microbiota ecology, but also that the fecal microbiota in Angus steers is more easily influenced by heat stress in toxic tall fescue grazing steers versus those on non-toxic tall fescue pastures [69]. This could have multiple origins, one being impaired gut wall integrity. Of note, Alrashedi [121] found increased ruminal *Firmicutes* and decreased fecal *Bacteroidetes* in ewes grazing high and medium endophyte-infected tall fescue pastures, with ruminal *Prevotella* and fecal *Coriobacteriaceae* operational taxonomic units (OTUs) associating with body weight changes. Finally, Koester, et al. [122] reported significantly decreased bacterial and fungal fecal diversity and richness in Angus cattle that had low tolerance to toxic tall fescue exposure. In this study, the *Neocallimastigaceae* family was increased in high tolerant steers, whereas the genus *Thelebolus* was increased in lower tolerance steers. Interestingly, this study also reported farm-to-farm variability in the fecal microbiota of Angus cattle, indicating that putative fecal biomarkers should be systematically evaluated to ensure cross-study validation. Overall, these data affirm that ergot alkaloids/toxic tall fescue significantly alter gut physiology and microbiota along the enteric tract. However, more in vivo analyses need to be performed to understand the importance of the microbiota in fescue toxicosis adaptation/development and whether any of the potentially adverse effects could be targeted therapeutically at gut level. 

#### 5.2.2. Metabolic Effects of Toxic Tall Fescue Grazing

For a comprehensive review on the overarching physiological changes associated with fescue toxicosis, the authors would direct readers to Strickland, et al. [123]. Briefly, most cellular components of the blood are not affected by toxic tall fescue grazing, but decreased erythrocyte size and hemoglobin content have been reported [124]. One consistent finding in studies analyzing the effects of toxic tall fescue on blood parameters is decreased circulating cholesterol and triglyceride levels [86,124]. Interestingly, bolus injection with ergotamine decreases plasma insulin, while increasing plasma glucagon concentrations within 1 h post-injection [125]. The impact of ergot alkaloids on plasma cortisol, another metabolism regulating hormone, levels are variable [86,126]. Cattle consuming toxic tall fescue have large variations in circulating norepinephrine and epinephrine levels, which may be associated with altered behavior [127,128]. Tall fescue affects circulating metabolites and hormones and both intra- and extra-hepatic enzyme activities [86], indicating global metabolic effects. Jackson, et al. [129] analyzed specific blood analytes in steers on either high or low endophyte-infected tall fescue pastures, but we first utilized untargeted high-resolution metabolomics to assess global biochemical changes that result from toxic tall fescue grazing [68]. Both plasma and urine metabolomes, namely amino acid and glycerophospholipid metabolism [68], were among the major effects. Interestingly, some metabolic effects are modulated by hot and humid environmental conditions [69], highlighting the complexity of fescue toxicosis pathophysiology. While this work is a starting point, to expand upon these initial results, substantial additional efforts that assess global metabolic changes in response to *E. coenophiala* infection or exposure, in the fescue plant and grazing animal, are needed.

#### 5.2.3. Understanding the Fescue Toxicosis Integrome

**Figure 2:**
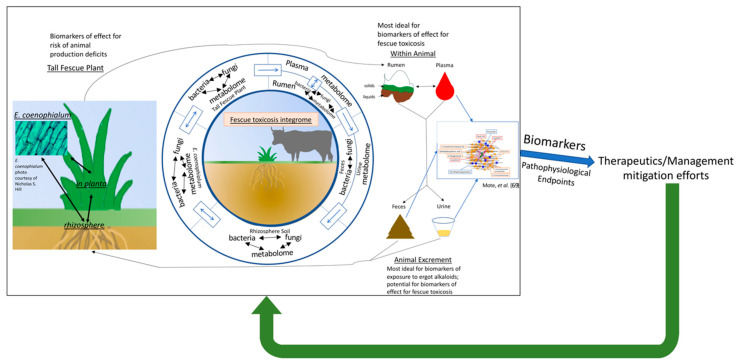
A schematic of the fescue toxicosis integrome. The integrome begins by understanding the complex relationship between the rhizosphere microbiota and metabolome, tall fescue plant microbiota and metabolome, and how these are influenced by the endophyte and grazing stresses. Next, endophyte-related changes are integrated into the animal physiology through microbiota or metabolic changes in the rumen that are reflected by metabolic changes in the plasma prior to microbiota and metabolome effects at the excretion level (feces and urine). The integrome will also allow for understanding the potential for feedback regulation of plant physiology and plant-endophyte relationship by components of the animal excrements, alongside grazing stresses. Of note, changes on the plant side have the potential to be used as biomarkers that have the utility of predicting the risk of animal production deficits of fescue toxicosis. Further, while blood tissues and rumen samples are most suitable for finding biomarkers of effects, animal excrements are ideal for identifying biomarkers of exposure (possibly biomarkers of effects as well) because of their ease of access. One example from our previous paper [69] shows how top-down strategies, while centering of global effects, can also identify biological features (i.e., OTUs and metabolic features) that associate with pathophysiological effects (e.g., animal weight gains), highlighting their utility as biomarkers. Biomarkers and pathophysiological endpoints can be used, in conjunction with other methods, to identify potential new and/or improved fescue toxicosis mitigation approaches. They will be then evaluated for their efficacy at multiple levels and the outcomes of the evaluation will inform stakeholders on best ways to improve disease management. Similar approach can be adapted to other conditions, including other economically important toxicoses.

Integrating microbiota and metabolomics data will help outline the relationship between the microbiota, global metabolism, and other parameters such as animal performance. For example, it is well known that shifts in the microbiota influence the presence of microbial metabolites that, in turn, modulate other downstream processes. Toxic tall fescue grazing alters metabolism; however, the microbiome, metabolome and other important physiological parameters have generally been investigated in isolation and not in an integrative manner despite their interconnectedness. The fescue toxicosis integrome is complex; although the plant-endophyte-animal relationship has not been evaluated, we have begun to move in this direction. We successfully integrated the plasma and urine metabolomes with fecal microbiota data to interrogate changes in the microbiota-metabolome relationship that occur after toxic tall fescue exposure [69]. Within this study, the bidirectional relationship between the microbiota and metabolome [130,131], and how this shifts after placement on toxic versus non-toxic tall fescue pastures, was investigated. To do this, we used sparse partial least squares regression (sPLS) to integrate the datasets followed by a differential network analysis to reveal a highly correlated network of fecal OTUs and plasma/urine metabolites. We then identified the OTUs that were correlated with parameters of interest (e.g., average daily weight gains) and found a subnetwork that provides potentially valuable therapeutic targets. As highlighted in Figure 2, understanding the plant-endophyte relationship and how changes in the plant influence bovine physiology through integrating plant and animal data provides a framework for understanding how additional stressors or geographical/cultivar change the disease.

## 6. Where Is This Approach for Fescue Toxicosis Research Headed?

Currently, it is increasingly apparent that indirect, monoamine receptor-independent mechanisms play an important role in fescue toxicosis pathogenesis. To evaluate these, we propose a framework that can be used for interrogating the fescue toxicosis integrome. Understanding the soil-plant-endophyte relationship and how altering this relationship could then influence the toxic fescue grazing beef is a necessary first step (Figure 2). Next, integrating these data with the animal response data (e.g., the rumen/fecal microbiota and metabolomes) will pinpoint some important features that could be targeted therapeutically at the plant level, or as other (i.e., non-ergot alkaloid) potential biomarkers that predict animal production deficit risk (Figure 2). This approach can also point to where pathophysiological changes begin, what other processes may be directly/indirectly influenced within the animal and explain systemic effects of fescue toxicosis (Figure 2). Finally, what the animal is exposed to, as a result of toxic tall fescue grazing will influence host physiology, and ultimately, what that animal excretes. Consequently, grazing animal excrements and grazing stresses will affect the tall fescue plant/endophyte physiology. The integrome considers how these changes, in combination with other environmental factors, influence what the animals are exposed to (Figure 2). Animal biological matrices, such as tissues or blood, have a higher barrier to accessibility, but still provide utility for identifying biomarkers of effects resultant from sensitivity to physiological (whether GI or systemic) changes (Figure 2). The urine or fecal matter provide unique opportunity to identify biomarkers (metabolites or microbes [OTUs]) of exposure to ergot alkaloids and toxic tall fescue, as they are easily accessed (Figure 2). These two readily accessible biological matrices would be ideal for biomarkers of effect as well, if a urine/fecal change is reflective of systematic effects. We used a similar approach to begin investigating the integrome, where we integrated plasma/urine high-resolution metabolomics and fecal bacterial microbiota data to investigate the microbiota-metabolome interactions [69]. Ultimately, we identified one small cluster of metabolites anchored by bacterial OTUs of interest. One *Ruminococcaceae*, one *Clostridium*, and one *Peptococcaceae* candidate genus *rc4-4* were highlighted within this network, as they were highly correlated with physiological endpoints of interest (i.e., animal weight gains, urinary ergot alkaloids), plasma (e.g., 4,8-dihydroxyquinoline) and urine (e.g., 18-oxocortisol) metabolites, and were present in the networks under both thermoneutral and hot and humid environmental conditions. Therefore, these metabolites could be useful as biomarkers of a hindgut microbiota associated with signs of fescue toxicosis pathophysiology (i.e., reduced animal productivity) under most ambient environmental conditions [69]. Expanding upon this by integrating the foregut and plant information will be an important step forward towards deciphering the fescue toxicosis integrome. 

One of the other most important issues moving forward will be ensuring analytical and computational pipelines are comparable. For example, not only should researchers include sequencing depth and coverage for NGS-based genomics; it was recently shown that differing preprocessing (i.e., Mothur vs QIIME) methods of NGS data can influence the abundance profiles of less common microbes of the rumen [132]. Further, recent comparisons between pipelines leading to amplicon sequence variants (ASVs) and OTUs seem to indicate some differences in specificity between the two, with different OTU-based pipelines producing some non-overlapping spurious OTUs [133]. For metabolomics, differences in analytical platforms, computational methods, and database matching algorithms for metabolomics could also result in metabolic feature variability [134,135,136,137]. Finally, while still a less visible, yet budding field, multi-‘omics integration methodologies, such as xMWAS [21], can vary, and their continual development and improvement will increase consistency. Overall, while this integrative approach will be a great complement to classical techniques and provide additional context for ongoing fescue toxicosis research, much progress still needs to be made to ensure its proper implementation; the current framework could be helpful as an initial guideline.

## 7. Applicability of Integrative Interactomics across Agriculture

While the latter portion of this review focused on developing a framework for using integrative interactomics to help interrogate fescue toxicosis’ pathophysiology from a multi-level perspective, this concept is widely applicable across the agricultural sector. The influence of the microbiota on animal production and in numerous disease states, as well as use of metabolomics to identify potential biomarkers of interest, is increasing. For example, Kumar, et al. [138] proposed similar systems-biology based techniques as a potential route for smarter agricultural innovation based on the idea that these techniques help link genotype and phenotype. Shameer, et al. [139] took it a step further by emphasizing using these techniques to develop sustainable agricultural systems, an important and timely endeavor. From an animal perspective, as stated before, these systems biology techniques have been used to assess animal productivity, more so in dairy cattle [140,141]. Although no study has comprehensively evaluated what we have termed the integrome, the idea of integrating ‘omics data sets has been previously proposed as a potential tool from a meat science perspective [142]. To provide an analogous example of how this could be useful as a complementary approach to classical methods for fescue toxicosis, Johne’s Disease, a bovine disease that culminates with weight loss, is used. Recent investigation found that Johne’s Disease is associated with profound fecal microbiota dysbiosis [143]. Using this framework could help investigators to detect downstream processes that are affected by gastrointestinal dysbiosis induced by Johne’s Disease, select ones that track with wasting and muscle loss, and identify possible new therapeutic target(s). Moreover, it is well known that many mycotoxins are detrimental to livestock productivity, whether through reproductive inefficiencies or by reducing weight gains [144]; this framework is readily applicable towards any mycotoxin, highlighting the translatability of this approach across farm animal exposures and diseases. 

## 8. Conclusions

In this review, the case for integrative interactomics as the next step in toxicology and animal agriculture is presented. It should be clear that studying the integrome will be a widely adaptable field moving forward as we continue to improve methodologies. For fescue toxicosis, the proposed framework of the fescue toxicosis integrome is an important step forward. As high-throughput techniques to evaluate different aspects of how toxic tall fescue grazing influences livestock physiology become a mainstay, providing a framework can help align the field towards a cohesive, integrative approach to this complex problem. Taking such an approach will allow identification true fescue toxicosis pathophysiological outcomes, which can help address some study-to-study variations. Using the outcomes from integrative interactomics analyses in conjunction with key production and/or pathophysiology indices will lead to therapeutically valuable target(s) identification and, ultimately, better disease management. Overall, adopting an integrome-based framework, like the one proposed here, has the potential to benefit animal agriculture moving forward.
